# Molecular markers for tracking the origin and worldwide distribution of invasive strains of *Puccinia striiformis*

**DOI:** 10.1002/ece3.2069

**Published:** 2016-03-20

**Authors:** Stephanie Walter, Sajid Ali, Eric Kemen, Kumarse Nazari, Bochra A. Bahri, Jérôme Enjalbert, Jens G. Hansen, James K.M. Brown, Thomas Sicheritz‐Pontén, Jonathan Jones, Claude de Vallavieille‐Pope, Mogens S. Hovmøller, Annemarie F. Justesen

**Affiliations:** ^1^ Department of AgroecologyAarhus UniversityFlakkebjergDK‐4200SlagelseDenmark; ^2^ Institute of Biotechnology & Genetic EngineeringThe University of Agriculture, Peshawar25000PeshawarPakistan; ^3^ The Sainsbury LaboratoryNorwich Research ParkNorwichNR4 7UHUK; ^4^ ICARDARegional Cereal Rust Research CentreAegean Agricultural Research Institute P.K. 9Menemen/İZMİRTurkey; ^5^ National Institute of Agronomy of Tunisia (INAT)Avenue Charles Nicolle43 TN‐1082 El MahrajèneTunisia; ^6^ INRA UMR 320 Génétique VégétaleFerme du MoulonF‐91190Gif sur YvetteFrance; ^7^ John Innes CentreNorwich Research ParkNorwichNR4 7UHUK; ^8^ Center for Biological Sequence AnalysisDepartment of Systems BiologyTechnical University of DenmarkBuilding 208DK‐2800Kongens LyngbyDenmark; ^9^ INRA UR 1290 BIOGER‐CPPBP01F‐78850Thiverval‐GrignonFrance; ^10^ Present address: Eric Kemen Max Planck Institute for Plant Breeding ResearchCarl‐von‐Linné‐Weg 1050829CologneGermany

**Keywords:** Aggressive strain, PCR‐based markers, plant pathogen, wheat yellow rust/stripe rust

## Abstract

Investigating the origin and dispersal pathways is instrumental to mitigate threats and economic and environmental consequences of invasive crop pathogens. In the case of *Puccinia striiformis* causing yellow rust on wheat, a number of economically important invasions have been reported, e.g., the spreading of two aggressive and high temperature adapted strains to three continents since 2000. The combination of sequence‐characterized amplified region (SCAR) markers, which were developed from two specific AFLP fragments, differentiated the two invasive strains, *Pst*S1 and *Pst*S2 from all other *P. striiformis* strains investigated at a worldwide level. The application of the SCAR markers on 566 isolates showed that *Pst*S1 was present in East Africa in the early 1980s and then detected in the Americas in 2000 and in Australia in 2002. *Pst*S2 which evolved from *PstS1* became widespread in the Middle East and Central Asia. In 2000, *Pst*S2 was detected in Europe, where it never became prevalent. Additional SSR genotyping and virulence phenotyping revealed 10 and six variants, respectively, within *PstS1* and *PstS2,* demonstrating the evolutionary potential of the pathogen. Overall, the results suggested East Africa as the most plausible origin of the two invasive strains. The SCAR markers developed in the present study provide a rapid, inexpensive, and efficient tool to track the distribution of *P. striiformis* invasive strains, *PstS1* and *PstS2*.

## Introduction

Invasive species, genotypes and/or specific strains of microorganisms may pose a serious threat to the stability of ecosystems and increase the fluctuations in crop productivity (Palm 2001; Lee 2002; Hodson 2011). The increasing number of invasive crop pathogens, which has been reported in the recent past, is thereby contributing to reduce food security in general (Parker and Gilbert 2004; Desprez‐Loustau et al. 2007). The development of risk‐assessment and management strategies for food production relies on knowledge about origin, migration routes and distribution of threatening strains of plant pathogens (Campbell 2001; Perrings et al. 2002), which require efficient tracking and monitoring systems. In case of crop pathogens which may spread rapidly and across long‐distances, research and monitoring should address pathogen variability at continental or global scales.

The inconspicuous nature of many crop pathogens makes the application of molecular markers and population genetic analyses highly valuable (McDonald 1997; Gladieux et al. 2008; Hovmøller et al. 2008; Ali et al. 2014b). Their application in different populations and environments may provide the basis for further development of markers targeting specific strains and/or essential epidemiological features (Carvalho et al. 2001; Hodson et al. 2012; Zamor et al. 2012). Development and application of such markers have proven to be highly valuable for the efficient detection and tracking of invasive strains/species and could be applied to a wide range of microbial invasive species like fungi (Kroon et al. 2004; Hodson et al. 2012), viruses (Brown 2000), bacteria (Carvalho et al. 2001) and algae (Zamor et al. 2012). Several molecular marker techniques have been exploited to understand microbial population biology e.g., Random Amplified Polymorphic DNA (RAPD), Restriction Fragment Length Polymorphism (RFLP), Amplified Fragment Length Polymorphism (AFLP), Simple Sequence Repeats (SSR) and Single Nucleotide Polymorphism (SNP) markers (McDonald 1997). The AFLP markers, which were widely used in the 1990s and 2000s to detect genetic variation in populations with very low genetic diversity (e.g., Justesen et al. 2002; Enjalbert et al. 2005) can be converted into sequence‐characterized amplified region (SCAR) markers (Paran and Michelmore 1993). The codominant, single‐locus SCAR markers allow a quick and easy PCR amplification‐based detection of the defined fungal strains (Hermosa and Grondona 2001; Naeimi and Koscsubé 2011). Application of SCAR markers facilitates testing of a large number of isolates and could be useful to track the origin and spread of microbial pathogens with long distance dispersal capacity and invasion potential.

The wheat yellow/stripe rust pathogen, *Puccinia striiformis* can undergo long distance dispersal and has caused numerous invasions (Zadoks 1961; Wellings and McIntosh 1990; Markell and Milus 2008; Hovmøller et al. 2015). While the recently suggested centre of diversity of *P. striiformis* is in the Himalayan and near‐Himalayan region (Ali et al. 2010, 2014a,b; Thach et al. 2016), the pathogen is distributed worldwide and is often associated with severe economic losses (Stubbs 1985; Hovmøller et al. 2002, 2011; de Vallavieille‐Pope et al. 2012). Losses can be especially high when the epidemics are associated with exotic strains or populations, as these are rarely considered in breeding for disease resistance locally (Chen et al. 2002; Wellings 2007). Several cases of economically important incursions have been reported for *P. striiformis* but only very recently the origin of these were confirmed (Ali et al. 2014a; Hovmøller et al. 2015). In the early 20th century, the pathogen was reported for the first time in North and South America (Carleton 1915; Rudorf and Job 1934), most likely spreading from NW Europe (Hovmøller et al. 2011; Ali et al. 2014a). It was introduced accidentally in Australia in 1979 from NW Europe (Wellings and McIntosh 1990; Hovmøller et al. 2008) through human transmission (Wellings 2007). The strains first detected in South Africa in 1996 were later shown to be genetically related to populations in the Middle Eastern and Mediterranean regions, possibly spread by wind (Boshoff et al. 2002; Hovmøller et al. 2008; Ali et al. 2014a). Apart from these recent incursion events in previously noncolonized areas, *P. striiformis* has been important in the context of invasion and recolonization through emergence of new races and strains. For example, virulence to the resistance gene *Yr9* was detected in races in East Africa (Ethiopia) in 1986, and the same virulence was in subsequent years observed in the Middle East and Indian subcontinent and thereby seriously affecting *Yr9*‐resistant wheat varieties across large areas for more than a decade (Singh et al. 2004). Since 2000, the emergence of two high temperature‐adapted aggressive strains, *PstS1* and *PstS2*, resulted in geographical expansion of *P. striiformis* epidemics into Western Australia and the South Eastern USA, where the disease had not previously been considered a problem (Chen 2005; Milus et al. 2009). Since 2011, invasive strains of the “Warrior” and “Kranich” races have largely replaced the pre‐existing NW European populations (Hovmøller et al. 2015; Hubbard et al. 2015).

*PstS1* and *PstS2* were clearly distinct from the local pre‐2000 populations of North America, Australia and Europe (Hovmøller et al. 2011) and representative isolates of these were shown to be more aggressive and adapted to high temperatures than typical isolates from Europe and North America (Markell and Milus 2008; Milus et al. 2009). *PstS1* resulted in *P. strii‐formis* epidemics in the south‐central USA in 2000 and following years (Chen 2005; Milus et al. 2006) and in Western Australia in 2002 (Wellings et al. 2003). *PstS2* was reported in NW Europe with similar aggressiveness and strong differentiation from local *P. striiformis* populations but it did not have an adverse economic impact because most European wheat varieties were resistant to this strain (Mboup et al. 2012; Hovmøller et al. 2015). *PstS2* was also present in the Mediterranean region and the Middle East‐Red Sea area. Although both strains were related to a genetic group prevalent in Western Asia, North Africa and the Red Sea Area (Ali et al. 2014a), the exact geographical origin and spread of the two strains remain unknown, and the monitoring of further spread of these two strains would greatly benefit from the development of efficient and easy‐to‐apply diagnostic tools.

*PstS1* and *PstS2* differed from each other by only two polymorphic AFLP fragments and from other strains by at least 14 AFLP markers (Hovmøller et al. 2008). In this study we converted two AFLP markers into SCAR markers; P19M24.225 being specific to both *PstS1* and *PstS2* and distinguishing these from all other strains investigated; and P12M26.150 which was present in *PstS1* and other isolates and thereby distinguishing *PstS2* isolates from all others considered in the study (Hovmøller et al. 2008). Conversion of these AFLP bands into strain specific SCAR markers should provide a valuable and simple monitoring and tracking tool. The present study was designed (1) to develop PCR based SCAR markers for rapid and efficient detection of the two invasive strains *PstS1* and *PstS2,* (2) to assess the distribution of the two strains on a worldwide scale using these SCAR markers, and (3) to detect variation within the strains and to infer potential origin of these by comparing SSR genotypes and virulence phenotypes of *PstS1* and Pst*S2* with the worldwide genetic grouping (Ali et al. 2014a) and the virulence phenotypes of historical isolates from the “Stubbs collection” (Thach et al. 2016).

## Materials and Methods

### Selection and virulence assessment of isolates

A set of 566 isolates of *P. striiformis* were selected from a worldwide set of more than 5000 isolates available at BIOGER‐CPP, INRA, France and the Global Rust Reference Center (GRRC) at Aarhus University, Denmark. Isolates were selected primarily from year 2000 and onwards (525 of 567 isolates; Table 1) when the invasive *P. striiformis* strains were first detected (Milus et al. 2006; Hovmøller et al. 2008). Isolates were sampled by either the authors or their international collaborators from local field trials, trap nurseries or commercial fields as single lesions on detached wheat leaves (Table 1). The virulence phenotype of *P. striiformis* isolates was determined using differential cultivars and additional varieties (Hovmøller and Justesen 2007; Thach et al. 2015).

**Table 1:** Distribution of *Puccinia striiformis* isolates from worldwide representative geographical regions sampled over recent years to investigate the worldwide distribution and origin of the two invasive strains *PstS1* and *PstS2*

Geographical region	Country	Pre‐2000 samples		Post‐2000 samples	
		No. of isolates tested	Sampling year	No. of isolates tested	Sampling year
South Asia	Afghanistan	–	–	16	2009, 2010, 2011
	Nepal	–	–	19	2005, 2008
	Pakistan	–	–	21	2004, 2006, 2010, 2011
East Africa	Eritrea	–	–	18	2002, 2003, 2004, 2005, 2011
	Ethiopia	3	1977, 1986, 1987	35	2007, 2010
	Kenya	10	1982, 1986, 1989, 1991	15	2009, 2011
	South Africa	1	1996	0	–
Central Asia	Kazakhstan	–	–	6	2003
	Kyrgyzstan	–	–	6	2003
	Tajikistan	–	–	12	2010, 2011
	Uzbekistan	–	–	8	2003, 2010, 2011
Middle East	Azerbaijan	–	–	29	2005, 2009, 2010
	Iran	–	–	19	2005, 2011, 2012
	Iraq	–	–	8	2010, 2011
	Israel	–	–	6	2005, 2006
	Lebanon	3	1974, 1975, 1985	7	2006, 2012
	Saudi Arabia	1	1976	0	–
	Syria	1	1991	16	2004, 2009, 2010
	Turkey	1	1973	17	2005, 2011, 2012
	Yemen	–	–	19	2003, 2005, 2007
North Africa	Algeria	–	–	11	2005, 2006
	Morocco	–	–	25	2006, 2009
	Tunisia	–	–	10	2005, 2006, 2007
Europe	Austria	–	–	3	2000
	Cyprus	–	–	8	2006
	Denmark	5	1994, 1995, 1997	64	2001, 2002, 2003, 2004, 2006, 2009, 2010, 2011
	France	3	1986, 1989, 1997	2	2004, 2011
	Germany	–	–	12	2000, 2010, 2011
	Italy	–	–	9	2005, 2006
	Netherlands	1	1962	0	–
	Portugal	–	–	4	2006
	Spain	–	–	8	2006, 2012
	Sweden	–	–	38	2009, 2010, 2011
	United Kingdom	5	1975, 1978, 1991, 1995, 1998	13	2010, 2011
North America	Mexico	–	–	3	2002, 2003
	USA	6	1980, 1981, 1992, 1993, 1994, 1997	5	2000, 2003
South America	Argentina	–	–	1	2010
	Brazil	–	–	6	2010
	Chile	–	–	8	2010
	Uruguay	–	–	13	2010
Australia	Australia	1	1985	5	2002, 2004
Overall population		41	1962–1999	525	2000–2012

Using the published virulence phenotype (Hovmøller et al. 2008; Milus et al. 2009) for invasive strains *PstS1* and *PstS2* as selection criteria, virulence profiles of *P. striiformis* isolates from the historic Stubbs collection (Thach et al. 2015), comprising 2708 unique wheat yellow rust isolates sampled on six continents in 66 countries between 1958 and 1995 were inspected in order to identify historic samples with virulence profiles similar to those of *PstS1* and *PstS2*. A subset of these isolates from the Stubbs collection (Thach et al. 2015), was selected for genotyping with the newly developed SCAR markers (Table 1). Worldwide race groups were inspected at the database www.wheatrust.org using the “yellow rust” and “pathotype by country” and “race groups” selections.

### Isolation of genomic DNA

Genomic DNA was extracted either from 5 to 10 mg of urediniospores (dikaryotic) using a modified CTAB protocol (Enjalbert et al. 2002; Hovmøller et al. 2008) or from wheat leaf segments bearing a single lesion (Ali et al. 2011). Extracted DNA was quantified on an agarose gel or with the NanoDrop 2000 spectrophotometer (Thermo Fisher Scientific Inc., Waltham, MA, USA) and stored at −20°C.

### Purification and sequencing of AFLP bands

The two AFLP markers P19M24.225 and P12M26.150 (Hovmøller et al. 2008), were excised for selected positive isolates with a scalpel from AFLP gels that were blotted onto Whatman^™^ paper and aligned with the corresponding autoradiogram. Excised AFLP bands were dissolved in Tris‐EDTA pH 8.0 by incubation at 60°C for 2 h and the resulting DNA solution was collected by centrifugation at 12000 rcf for 20 min. The respective AFLP fragments were amplified with primers PstI+O and MseI+O (Vos et al. 1995; Justesen et al. 2002) in a Thermocycler (Eppendorf, Hamburg, Germany) using PCR conditions consisting of 94°C for 2 min 30 sec and 40 cycles of 94°C for 30 sec, 56°C for 1 min, and 72°C for 1 min, and a final extension at 72°C for 5 min. Standard PCR was performed in total reaction volume of 25 *μ*L containing the Eppendorf Taq DNA polymerase kit (Eppendorf) and 2.5 mM of dNTPs and using 15 *μ*L DNA solution.

PCR products were either cloned into the vector pGEM‐T (Promega, Fitchburg, WI, USA) according to the manufacturer's instructions or purified from an agarose gel with the QiaexII gel extraction kit (Qiagen, Hilden, Germany) prior to sequencing (Macrogen Europe, Amsterdam, The Netherlands) with primer pairs T7/SP6 or PstI+O/MseI+O respectively. Using Geneious^®^ Pro 6.1.4 software (Biomatters Ltd., Auckland, New Zealand), the obtained sequences were trimmed for low quality and vector traces after BLAST search against the NCBI VecScreen database as well as for artificially included primer sequences of PstI+O and MseI+O and were manually corrected for sequence read errors after multiple MUSCLE alignment (integrated in Geneious^®^ Pro 6.1.4; Biomatters Ltd.) of all homologous sequences. Identity of amplified sequences with respective AFLP markers was confirmed by alignment with the respective AFLP primers (Justesen et al. 2002).

### Development of PCR markers for invasive *P. striiformis* strains

Genomic sequences flanking the sequenced AFLP fragments were obtained by BLASTn search against (1). the sequence read archive, whole‐genome shotgun contigs, and transcriptome shotgun assemblies available for *P. striiformis* at GenBank; (2). *Puccinia* Group transcripts and genomic sequences of the *Puccinia* Group Sequencing Project, Broad Institute of Harvard and MIT (http://www.broadinstitute.org/); and (3). a shotgun genome assembly for the European *P. striiformis* isolate GB75/30 generated at The Sainsbury Lab, Norwich, United Kingdom (available on request from GRRC). Primers for sequencing were designed from identified flanking genomic regions to allow amplification and subsequent sequencing from both isolates harboring and lacking the respective AFLP fragment in order to identify precisely those nucleotide changes that account for the observed AFLP polymorphisms and to design allele specific PCR primers (Table 2). Respective genomic regions were amplified with these primers from DNA of selected isolates polymorphic for the respective AFLP marker and sequenced as described. SCAR markers detecting these polymorphisms were designed for both AFLP markers P19M24.225 and P12M26.150, and termed SCP19M24 and SCP12M26 respectively (SC: abbreviation for sequence characterized).

**Table 2:** Sequences of allele‐specific PCR and sequencing primers. Nucleotides in primers SCP19M24_a1R and SCP19M24_a2R that were artificially mutated for increased binding stringency are underlined

Marker name and allele (a)	Forward primer			Reverse primer			PCR product size (bp)	Application
	Name	Sequence 5′→3′	T_M_ (°C)	Name	Sequence 5′→3′ (artificially mutated nucleotides are underlined)	T_M_ (°C)		
SCP19M24 a1	SCP19M24_aF	GTAGAACTCTCACATTTTGTCCAT	56.5	SCP19M24_a1R	AGAATTCAGACTCATTAATCAAGTTACG	59.6	405	SCAR marker and sequencing PCR
SCP19M24 a2	SCP19M24_aF	GTAGAACTCTCACATTTTGTCCAT	56.5	SCP19M24_a2R	AAGAATTCACACTCATTAGTCAAGTTACA	59.6	385	SCAR marker and sequencing PCR
SCP12M26 a1	SCP12M26_a1F	CCTTCAAGAGATACTCTTTGATGTGG	58.6	SCP12M26_a1R	GTAGTGATGGTGTGGACTAGGCCTAA	59.9	491	SCAR marker
SCP12M26 a2	SCP12M26_a2F	AAATGGAGATTGAATCACGCG	60.4	SCP12M26_a2R	TATTGACCCAAACACCTCGTAAG	60.6	262	SCAR marker
SCP12M26 a1/a2	SCP12M26_seqF	GTATAGWGTAGGCGACTCCTTTGAG	59.8	SCP12M26_seqR	ATTGAGGGGCAATTCATCAG	59.9	1525 (a1) /1127 (a2)	Sequencing PCR

### Validation of SCAR markers

The SCAR markers developed in the study were validated against the seven representative isolates, which were originally used to define the two invasive strains (Hovmøller et al. 2008; Milus et al. 2009). The markers were further validated by comparing SCAR and AFLP results for all isolates in the study of Hovmøller et al. (2008). In case of disagreements between AFLP data for the two markers (Hovmøller et al. 2008) the polymorphic marker region was sequenced Macrogen Europe with the primers indicated in Table 2.

### Screening of worldwide *P. striiformis* collection to track *PstS1* and *PstS2*

The SCAR primers were then applied to a large set of 566 worldwide representative isolates to assess the geographical distribution of strains *PstS1* and *PstS2* (Table 1). PCR was performed in a total reaction volume of 20 *μ*L containing 1× GoTaq Flexi Buffer (5×; Promega), 1.5 mM MgCl_2_, 100 *μ*M of each dNTP, 1 *μ*M of each primer, 0.5 U GoTaq Flexi DNA polymerase (5 U/*μ*L; Promega) and 50 ng of genomic DNA. PCR conditions were 94°C for 2 min 30 sec and 35 cycles of 94°C for 30 sec, annealing at 60°C (for SCP19M24), or 63°C (for SCP12M26) for 1 min, and 72°C for 30 sec, and a final extension at 72°C for 5 min. PCR products were analyzed in 1.5% agarose gels.

### Microsatellite genotyping of *P. striiformis* isolates

To study diversity within the two strains from different geographical sampling areas and to infer their likely origin, microsatellite genotyping was performed for a subset of 131 isolates representing *PstS1* and *PstS2* strains. Microsatellite genotyping was done with a set of 16 SSR markers (RJN3, RJN4, RJN5, RJN6, RJN8, RJN9, RJN10, RJN11, RJN12, RJN13, RJO4, RJO18, RJO20, RJO21, RJO24, WU‐6), previously described (Ali et al. 2011) and used to describe the worldwide population structure (Ali et al. 2014a). These 16 SSRs were amplified in two multiplex reactions (Rodriguez‐Algaba et al. 2014). The number of multilocus genotypes (MLGs) among isolates classified as *PstS1* or *PstS2* via SCAR markers was identified using GENCLONE (Arnaud‐Haond and Belkhir 2007), and their profiles were compared with the SSR data of related multilocus genotypes (MLGs) detected in the worldwide populations by Ali et al. (2014a), particularly MLG‐99, which comprised *PstS1* and *PstS2* representative isolates and others with related virulence profiles. Genetic differentiation between *PstS1* and *PstS2* isolates was investigated by calculating F_ST_ values estimated with GENETIX v. 4.03 (Belkhir et al. 2004). The relationship of MLGs with the worldwide genetic groups also assessed through construction of a phylogenetic tree was based on neighbor‐joining using the software POPULATION (Langella 2008).

## Results

Two SCAR markers were developed for tracking the distribution and origin of the invasive *P. striiformis* strains, *PstS1* and *PstS2*. The two markers were validated in previously characterized isolates and then applied to a set of 566 isolates to track the origin and worldwide distribution of these two invasive strains.

### Strain specific markers, SCP19M24 and SCP12M26

Two AFLP markers (P19M24.225 and P12M26.150) were selected for cloning, resequencing, and development of SCAR markers. The marker P19M24.225 distinguished invasive strains *PstS1* and *PstS2* from all other *P. striiformis* isolates, and marker P12M26.150 distinguished *PstS2* isolates from all other isolates and hence allowed discrimination between *PstS1* and *PstS2* isolates.

**Figure 1:**
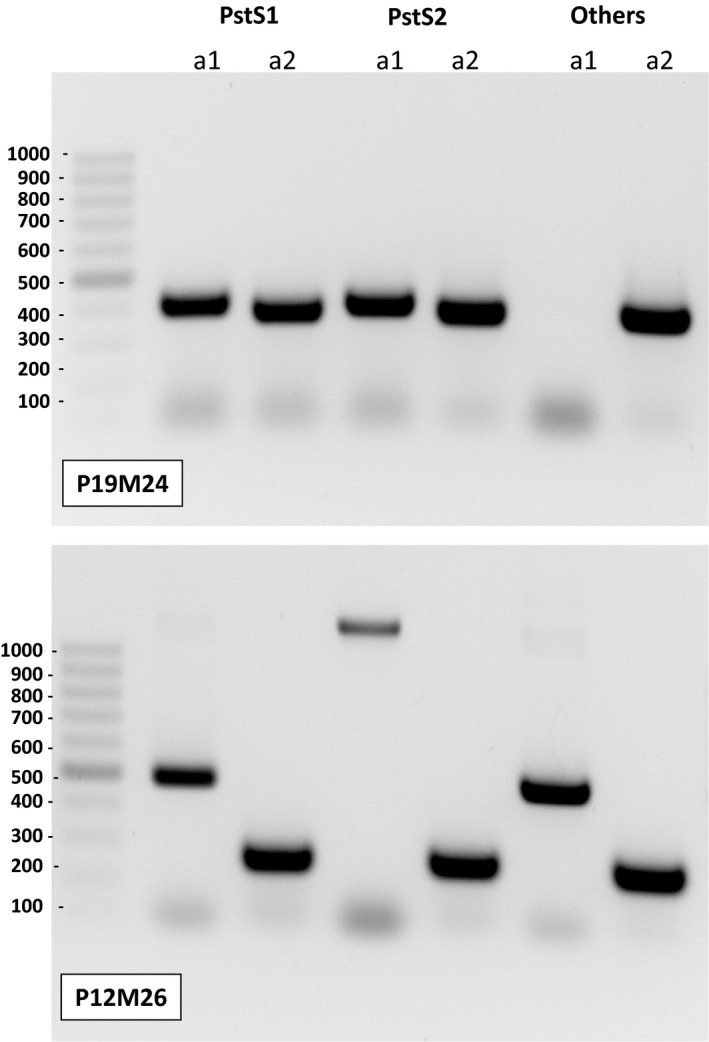
Gel electrophoresis depicting the PCR fragments generated after amplification of the SCAR markers SCP19M24 or SCP12M26 for representative isolates of *P. striiformis* invasive strains *PstS1* and *PstS2* and other isolates. Other isolates may also have lost the SCP12M26a1 fragment, however, these were not designated *PstS2* unless they also have the P19M24a1 fragment. In the figure a1 refers to allele 1 and a2 to allele 2.

The P19M24.225 AFLP fragment was cloned from the Danish *PstS2* isolate, DK80/01, and it was present in *PstS1* and *PstS2* only. Resequencing of the SCP19M24 SCAR region defined the length of the genomic sequence corresponding to the AFLP fragment as 199 bp in *PstS1* and *PstS2* isolates and 184 bp in other isolates. The AFLP variant in *PstS1* and *PstS2* isolates resulted from a SNP that created a new *Mse*I site, as well as an insertion of 15 bp within the AFLP fragment (Fig. S1). The SCAR primers detected the presence or absence of both the *Mse*I restriction site and the 15 bp insertion and yielded a 405 bp PCR product (SCP19M24a1) in *PstS1* and *PstS2* isolates but not in other isolates, and a 385 bp PCR product (SCP19M24a2) in all isolates (Fig. 1). Binding specificity was increased by inclusion of an artificial mutation of the third‐to‐last bp (from the 3′end) of the respective reverse primers for SCP19M24a1 and SCP19M24a2 (Table 2).

The absence of the P12M26.150 AFLP marker was generally specific to *PstS2* isolates, whereas *PstS1* and all other isolates had this marker. The P12M26.150 AFLP fragment was cloned from the noninvasive Pakistani isolate PK09/04. Cloning and resequencing of the P12M26.150 genomic region revealed that polymorphism was due to two single base pair deletions in *PstS1* and other isolates. Resequencing of the SCP12M26 SCAR region revealed two genomic sequences homologous to the AFLP fragment, one of 131 bp in all isolates (SCP12M26a2) and another (SCP12M26a1) of 129 bp in *PstS1* and other isolates but not in *PstS2* isolates (Fig. S2).

Owing to the high sequence similarity of the two length variants of this marker, SCAR primers were designed to detect sequence differences in the 5′ and 3′ flanking genomic regions (Fig. S2). The SCP12M26a1 SCAR primers (Table 2) amplified a product of 491 bp in *PstS1* and other isolates but not in *PstS2* isolates, and hence detected the *PstS2* strain (Fig. 1). The SCP12M26a2 SCAR primers (Table 2) amplified a 262 bp product in almost all isolates. The SCP12M26a1 SCAR primers do amplify in addition to the desired 491 bp product a band of 1156 bp from the a2 sequence due to high sequence similarity in the primer binding regions; however this band was not used for diagnostic purpose (Fig. 1).

### Validation of invasive strain specific markers

The results of tests of the SCAR markers SCP19M24 and SCP12M26 on the seven representative isolates that originally served to define invasive strains *PstS1* and *PstS2* were in agreement with the original classification (Table 3). For further validation, the SCAR markers were compared with AFLP data for another 155 isolates from a previous study. For marker SCP19M24, the two types of data were in full agreement (Table S1). Results for SCP12M26 agreed with AFLP results for 143 of 155 isolates (92.3%; Table S1). Resequencing of the SCAR marker region for the remaining 12 isolates, which all were sampled in Morocco and Syria in 2009, showed that they carried a mutation in the AFLP fragment. Altogether, these results indicated that the SCAR markers were reliable diagnostic markers for tracking the invasive strains *PstS1* and *PstS2*. While the two markers together enabled to detect the invasive strains, some isolates from Central and East Asia with a recombinant population structure had either acquired the SCP19M24a1 allele or lost the SCP12M26a1 allele.

**Table 3:** Confirmation of the *P. striiformis* invasive strain‐specific SCAR markers through their application to previously characterized isolates assigned to invasive aggressive and endemic nonaggressive groups

Isolate	Country	Sampling year	AFLP marker grouping (Hovmoller et al., 2008)	Aggressiveness test (Milus et al. 2009)	Interpretation based on SCAR markers	
					SCAR marker SCP19M24	SCAR marker SCP12M26
MT83	USA	1983	NW European group	Nonaggressive	Nonaggressive	Nonaggressive
Mex89.009	Mexico	1989	NW European group	Nonaggressive	Nonaggressive	Nonaggressive
AR90‐01	USA	1990	NW European group	Nonaggressive	Nonaggressive	Nonaggressive
DK16/02	Denmark	2002	NW European group	Nonaggressive	Nonaggressive	Nonaggressive
DK66/02	Denmark	2002	Strain 2	Aggressive	Aggressive	PstS2
E02/03	Eritrea	2003	Strain 2	Aggressive	Aggressive	PstS2
AR05‐IIG‐3	USA	2005	Strain 1	Aggressive	Aggressive	PstS1

### Worldwide distribution of the two invasive strains PstS1 and PstS2

After validation of the markers, they were applied to 566 isolates representative of worldwide populations, of which 41 were sampled pre‐2000 and 525 between 2000 and 2012. Either *PstS1* or *PstS2* were detected in all regions except South America (Table 4). In Australia, Mexico and USA only *PstS1* was detected. Both *PstS1* and *PstS2* were found in East Africa in Eritrea, Ethiopia and Kenya, with equal frequencies in Kenya and Ethiopia (Table 4). In South Asia and Middle East, North Africa, Central and Southern Europe only *PstS2* was detected at varying frequencies. Five isolates sampled in Kyrgyzstan, Afghanistan and Tajikistan, which represent areas where recombination is likely to occur, were diagnosed as *PstS1* with the SCAR test. However, the AFLP genotypes of these were clearly different from *PstS1*.

**Table 4:** Relative distribution of the two invasive strains (*PstS1* and *PstS2*) in worldwide populations of *P. striiformis*

Geographical origin	Country	Isolates tested	*PstS1*	*PstS2*	Other
South Asia	Afghanistan	16	–	2	14
	Nepal	19	–	–	19
	Pakistan	21	–	10	11
East Africa	Eritrea	18	–	7	11
	Ethiopia	38	10	10	18
	Kenya	25	11	11	3
	South Africa	1	–	–	1
Central Asia	Kazakhstan	6	–	–	6
	Kyrgyzstan	6	–	–	6
	Tajikistan	12	–	–	12
	Uzbekistan	8	–	2	6
Middle East	Azerbaijan	29	–	21	8
	Iran	19	–	15	4
	Iraq	8	–	7	1
	Israel	6	–	6	–
	Lebanon	10	–	3	7
	Saudi Arabia	1	–	–	1
	Syria	17	–	14	3
	Turkey	18	–	15	3
	Yemen	19	–	19	–
North Africa	Algeria	11	–	10	1
	Morocco	25	–	24	1
	Tunisia	10	–	9	1
Europe^a^	Austria	3	–	3	–
	Cyprus	8	–	8	–
	Denmark	69	–	1	68
	France	5	–	2	3
	Germany	12	–	4	8
	Italy	9	–	8	1
	Netherlands	1	–	–	1
	Portugal	4	–	4	–
	Spain	8	–	4	4
	Sweden	38	–	–	38
	UK	18	–	–	18
North America	Mexico	3	2	–	1
	USA	11	5	–	6
South America	Argentina	1	–	–	1
	Brazil	6	–	–	6
	Chile	8	–	–	8
	Uruguay	13	–	–	13
Australia	Australia	6	5	–	1
	Overall population	566	33	219	314

a *PstS2* isolates from Europe were selected according to virulence phenotype and are not representative for the European population.

**Figure 2:**
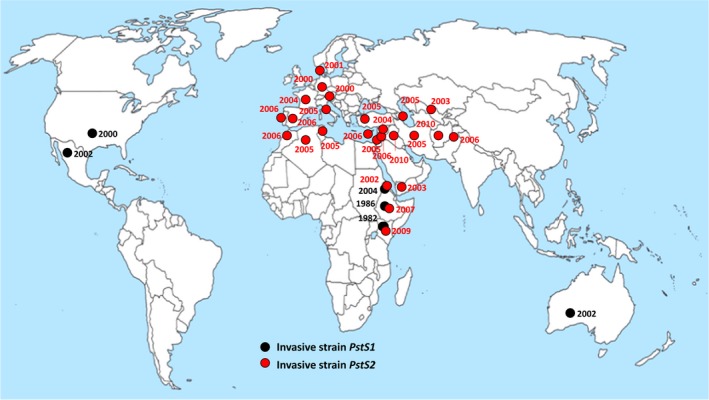
Worldwide incidence and years of first detection of the aggressive strains *PstS1* and *PstS2* of *Puccinia striiformis*, since the 1980s, as determined by genotyping with SCAR markers specific for *PstS1* and *PstS2*.

**Figure 3:**
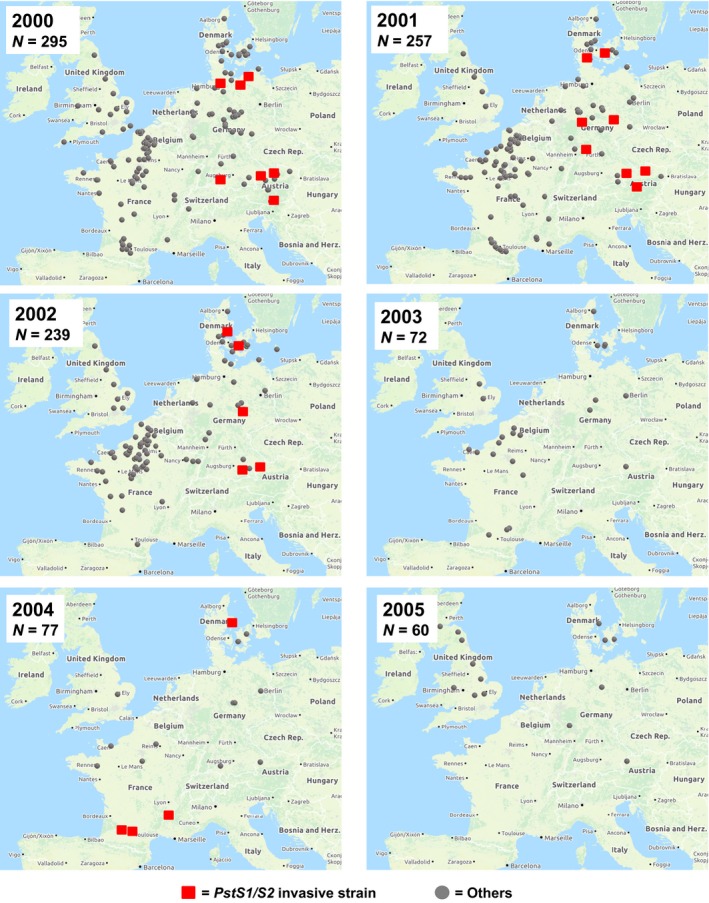
Prevalence of *P. striiformis* invasive strains *PstS2* across Europe since first appearance in 2000. Prevalence is determined by identifying isolates with virulence phenotypes typical of *PstS1* and *PstS2*.

Considering the temporal changes in the relative frequencies of the two invasive strains over years, *PstS1* was detected as early as 1982 and 1986 in East Africa, much earlier than *PstS2* which was first detected in 2000 in Europe (Fig. 2 and Table 4). While *PstS1* was only observed sporadically after 2000 outside the Americas and Australia, *PstS2* was detected in West and Central Asia in 2003 and then became prevalent in the Mediterranean area in 2005/2006 and in Middle East in 2005 and 2009. *PstS2* remained prevalent in Middle East and North Africa till 2012 (the latest year examined). In Europe, *PstS1* was not detected while *PstS2* never became prevalent in the native population (Fig. 3).

### Within strain microsatellite and virulence polymorphism

**Figure 4:**
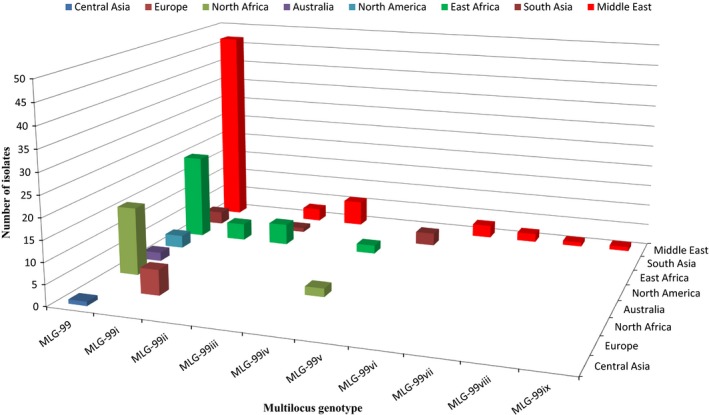
Geographical distribution of the 10 microsatellite multilocus genotypes detected within *P. striiformis* invasive strains *PstS1* or *PstS2*.

Microsatellite genotyping of 131 isolates of *PstS1* and *PstS2* revealed the presence of 10 distinct MLGs (Table 5). The most prevalent MLG, in the present study, designated MLG‐99 according to Ali et al. (2014a), was detected in both *PstS1* and *PstS2*. In this study MLG‐99 was detected in Australia, Central Asia, Middle East, East Africa and North Africa, and North America (Fig. 4). MLG‐99iv was also detected in both *PstS1* and *PstS2*. All the MLGs differed only at one locus from the MLG‐99, except MLG‐99vi, which differed at two loci. All the MLGs detected in *PstS1* and *PstS2* were assigned to the genetic group G4, the Middle East‐East African group (Ali et al. 2014a). There was no significant genetic differentiation between the *PstS1* and *PstS2* isolates as shown by the *F*_ST_ statistic value = 0.00083 (*P*‐value < 0.001).

**Table 5:** Number of resampled multilocus genotypes (MLGs) in *PstS1* and *Pst*S2 and their resampling in the worldwide *P. striiformis* population studied by Ali et al. (2014a)

MLGs detected	Resampling in the invasive strains		Resampling in worldwide population	Assignment to worldwide genetic group^a^	SSR loci differentiating from MLG‐1
	*PstS1*	*PstS2*			
MLG‐99	17	75	78	G4	–
MLG‐99i	–	10	–	G4	RJN‐11
MLG‐99ii	–	9	–	G4	RJO‐24
MLG‐99iii	–	6	–	G4	RJO‐24
MLG‐99iv	2	2	–	G4	RJN‐12
MLG‐99v	–	3	–	G4	RJO‐24
MLG‐99vi	–	3	–	G4	RJN‐12; RJO‐24
MLG‐99vii	–	2	–	G4	RJN‐9
MLG‐99viii	–	1	–	G4	RJN‐4
MLG‐99ix	–	1	–	G4	RJO‐21

a Assignment based on phylogenetic tree and group name according to Ali et al. (2014a).

The majority of isolates defined by SCAR markers as *PstS1* or *PstS2* had virulence corresponding to host resistance genes *Yr2, Yr6, Yr7, Yr8, Yr9,* and *Yr25* (Table 6). Additional virulence to *Yr27* was also common in the Middle East and Central Asia. Less frequent and often locally confined additional virulence was observed for *Yr1*,* Yr10,* and *Yr24*.

**Table 6:** Virulence phenotypes detected within *PstS1* and *PstS2* in a set of worldwide representative isolates

Virulence Phenotype^a^	Years of detection (sampling)	Present in strain
‐,2,‐,‐,‐,6,7,8,9,‐,‐,‐,‐,25,‐,‐,‐,AvS	2000, 2003, 2005, 2009, 2010, 2011	PstS1 and PstS2
‐,2,‐,‐,‐,6,7,8,9,‐,‐,‐,‐,25,27,‐,‐,AvS	2001, 2004, 2005, 2007, 2009, 2010, 2011	PstS1 and PstS2
‐,2,‐,‐,‐,6,7,8,9,10,‐,‐,24,25,‐,‐,‐,AvS	2004, 2005, 2009	PstS2
‐,2,‐,‐,‐,6,7,8,9,10,‐,‐,24,25,27,‐,‐,AvS	2010	PstS1 and PstS2
1,2,‐,‐,‐,6,7,8,9,‐,‐,‐,‐,25,‐,‐,‐,AvS	2009	PstS2
1,2,‐,‐,‐,6,7,8,9,‐,‐,‐,‐,25,27,‐,‐,AvS	2009	PstS1 and PstS2

a Figures and symbols designate virulence and avirulence(‐) corresponding to yellow rust resistance genes: Yr1, Yr2, Yr3, Yr4, Yr5, Yr6, Yr7, Yr8, Yr9, Yr10, Yr15, Yr17, Yr24, Yr25, Yr27, Yr32, and the resistance specificity of “Spalding Prolific” and “Avocet S”.

### Origin of the two invasive strains

**Figure 5:**
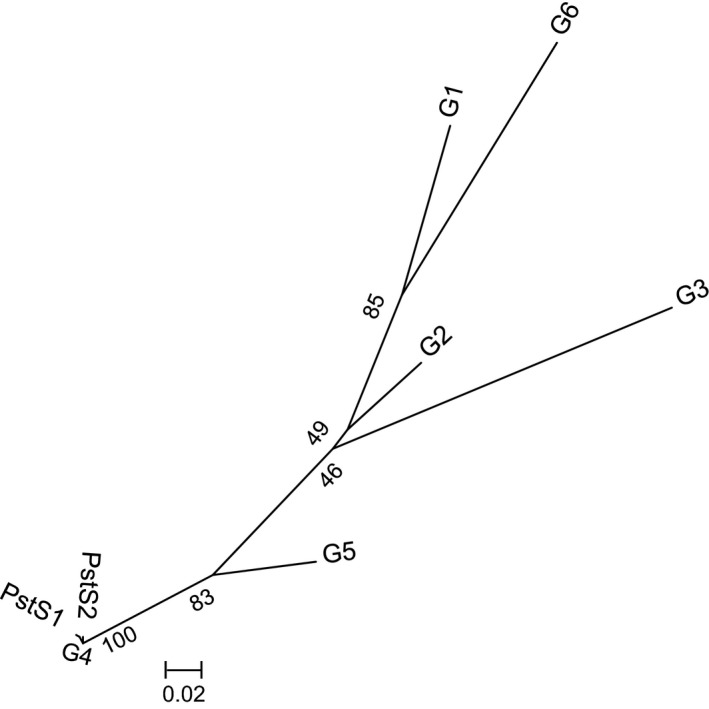
Neibor‐Joining phylogenetic tree based on microsatellite distance for 132 isolates representing *P. striiformis* invading strains (*PstS1* and *PstS2*) in comparison with the worldwide genetic groups of Ali et al. (2014a). G1 = China; G2 = Nepal; G3 = Pakistan; G4 = Middle East‐East Africa; G5 = Mediterranean region; and G6 = NW Europe.

The origin of the two invasive strains was inferred from their presence in temporally spaced populations, their virulence phenotype and microsatellite genotype in comparison with worldwide reference data. The virulence phenotype of 138 isolates from the historic Stubbs' collection sampled between 1958 and 1995, resembling the virulence phenotype of *PstS1* and *PstS2,* all originated from East Africa. The SCAR test of 13 of these revealed the presence of *PstS1* as early as 1982 in Kenya and in 1986 in Ethiopia (Fig. 2) but *PstS2* was not detected among these early samples (Fig. 2). The phylogenetic tree based on the microsatellite genotyping of a total of 132 *PstS1* and *PstS2* isolates, which showed no or limited divergence from isolates collected in the Middle East/East Africa, further supported an East African origin (Fig 5).

Virulence phenotype related to the strain *PstS1*/*S2* in the pre‐2000 Stubbs collection suggested that they first appeared in Kenya and subsequently in Ethiopia, Rwanda, Burundi, and Tanzania. Two variant virulence phenotypes were detected in the older East African isolates, compared to more recent invasive strains, differing from *PstS1* in virulence to *Yr3* or *Yr25* (data not shown).

## Discussion

The development of a simple, reliable, and easy‐to‐apply molecular diagnostic tool enabled to describe the worldwide distribution of the invasive *P. striiformis* strains *PstS1* and *PstS2* along with identification of its origin in East Africa.

### Invasive strain specific SCAR markers

The two SCAR markers were developed for rapid detection of invasive strains *PstS1* and *PstS2* in the *P. striiformis* population. The SCAR markers were conserved across samples of diverse geographical origin, as revealed through DNA sequencing of the SCAR fragments (data not shown). These SCAR markers are indicative of *PstS1* and *PstS2* in clonal populations of *P. striiformis* and are thus reliable and rapid diagnostic markers for tracking and monitoring of these aggressive invasive strains. They can be used on a worldwide scale to detect their arrival and prevalence to feed into prediction and early warning systems. However, diagnostic markers may become reassorted in the fungal genome in sexually reproducing populations in Central and Eastern Asia (Ali et al. 2014a), where the presence of SCP19M24 and the absence or presence of SCP12M26 does not necessarily indicate that an isolate belongs to *PstS1* or *PstS2*. In such recombinant populations, the use of these diagnostic markers could be coupled with microsatellite genotyping to confirm if the genotype is related to the MLGs typical of *PstS1* or *PstS2*. Molecular diagnostic markers to track invasive species and strains have been useful in a wide range of invasive microbial pathogens e.g., the invasive Ug99 lineage of rust fungus *P. graminis* f.sp. *tritici* (Hodson et al. 2012), invasive whitefly vector–*Begomovirus* complexes (Brown 2000), invasive and noninvasive bacteria *Campylobacter jejuni* and *C. coli* (Carvalho et al. 2001) and the invasive harmful algal bloom species *Prymnesium* (Zamor et al. 2012).

### Origin and worldwide spread of PstS1 and PstS2; application of SCAR markers

*PstS1* was first reported in 2000 in the USA and in 2002 in Australia while *PstS2* was first reported in 2000 in Europe and then in Africa and Asia (Chen et al. 2002; Hovmøller and Justesen 2007; Markell and Milus 2008). However, analyses of pre‐2000 isolates revealed the presence of *PstS1* as early as 1982 in Kenya, followed by the neighboring countries, Ethiopia in 1986, Rwanda and Burundi in 1988, and Tanzania in 1990. Moreover, *PstS1* was not detected outside East Africa until first appearance in North America and Australia (Hovmøller et al. 2008), and East Africa was the only region where both strains were detected. Finally, isolates which had been categorized as *PstS1*/*PstS2* by the SCAR test were all assigned the East African/Middle East genetic group based on microsatellite genotyping (Ali et al. 2014a). All these facts confirmed East Africa as the most likely origin of the two invasive strains.

With an origin in East Africa, *PstS1* would have spread further in the region and later on to North America and Australia. Mutation of *PstS1* into *PstS2* and its subsequent spread into the Middle East, North Africa and subsequently Europe resulted in their first detection in 2002 (Flath and Bartels 2002; Hovmøller and Justesen 2007; Hovmøller et al. 2008). In the post‐2000 worldwide populations, either *PstS1* or *PstS2* was present in all regions investigated. During their worldwide dispersal, the two strains diversified in terms of virulence and multilocus genotypes; at least 10 distinct MLGs were detected in *PstS1*and *PstS2*, though all were closely related to the Middle Eastern‐East African genetic group G4 (Ali et al. 2014a). The most prevalent multilocus genotype, identified in the present study corresponding to MLG‐99 in Ali et al.(2014a) was the most common MLG worldwide. Although virulences to the resistance genes *Yr2, 6, 7, 8, 9, 25* were characteristic of *PstS1* and *PstS2* (Milus et al. 2006; Hovmøller et al. 2008), further additional virulences would have been acquired by the strain during its spread and establishment.

The first appearance of *PstS1* in USA (in 2000) and Western Australia (in 2002) was followed by severe yellow rust epidemics in these areas (Chen et al. 2002; Milus et al. 2006; Wellings 2007). *PstS2* was first detected in West and Central Asia in 2003 (samples from earlier years were not available) and soon became prevalent in Middle East and North Africa, where it was found at the origin of major epidemics, see www.wheatrust.org. The high temperature adaptation of these two strains (Milus et al. 2006, 2009; Markell and Milus 2008) could have resulted in their establishment in warm climates, which were previously not considered to be conducive for *P. striiformis* (Milus et al. 2006; Markell and Milus 2008). The spread of these two invasive strains may be associated with the break‐down of the widely deployed *Yr9* resistance gene in the Middle East and South Asia in the 1980s and 1990s (Singh et al. 2004). The overall aggressiveness of the strains very likely contributed to their prevalence worldwide, particularly in the warmer climates of Middle East and North Africa.

In some regions, like Middle East and North Africa, these invasive strains (*PstS2* in this case) became dominant in the locally adapted *P. striiformis* populations and virulence phenotype data available at the database www.wheatrust.org suggests that these strains continue to affect wheat production. This has resulted in the shift in the population in Middle East and North Africa, where the pre‐2000 populations were strongly divergent from current populations (Bahri et al. 2009; Ali et al. 2014a; Thach et al. 2016). In other regions, like South Asia, they did not dominate the native *P. striiformis* population (Ali et al. 2014b,c). Although *PstS2* was detected in Europe in 2000 (Flath and Bartels 2002; Hovmøller et al. 2008), it never increased in frequency to dominate the native population of NW Europe (Hovmøller et al. 2015). This could be due to the lack of virulences to the resistance genes in widely deployed varieties in Europe (Hovmøller 2007; de Vallavieille‐Pope et al. 2012), as in the case of another Mediterranean strain, *PstS3* (Ali et al. 2014a), which never established in NW Europe due to the lack of certain virulences (Mboup et al. 2012; Hovmøller et al. 2015). This emphasizes the benefit of deploying crop varieties representing a diverse set of resistance genes to counteract the potential threat of invasive strains.

## Conclusions

The SCAR markers developed in the current study provide a rapid, inexpensive, and efficient tool to track the distribution of *P. striiformis* invasive strains, *PstS1* and *PstS2*. The study also revealed the distribution of the two strains across the world and identified East Africa as their origin. The worldwide spread and establishment of the two invasive strains reflect the adaptive potential of crop pathogens and the homogeneity in agricultural ecosystems, where genetically uniform crop varieties often are grown across large areas. The markers will enable further tracking of these strains, while the information in the study should encourage a better management of agro‐ecosystems in terms of resistance gene deployment to combat future invasion risks.

## Conflict of Interest

None declared.

## Supporting information


**Fig. S1.** Sequence alignment and primer binding sites for the polymorphic genome regions of SCAR marker SCP19M24.
**Fig. S2.** Sequence alignment and primer binding sites for the polymorphic genome regions of SCAR marker SCP12M26.
**Table S1.** Comparison of the performance of SCAR markers in relation to that of AFLP markers.
