# Supplementary material for: Molecular markers for tracking the origin and worldwide distribution of invasive strains of Puccinia striiformis
Source: Ecol Evol. 2016 Mar 20;6(9):2790–804. doi: 10.1002/ece3.2069 (PMC4800029; doi:10.1002/ece3.2069)
Supplement: Supplementary file 1 — Fig. S1. Sequence alignment and primer binding sites for the polymorphic genome regions of SCAR marker SCP19M24. Fig. S2. Sequence alignment and primer binding sites for the polymorphic genome regions of SCAR marker SCP12M26. Table S1. Comparison of the performance of SCAR markers in relation to that of AFLP markers. [file ECE3-6-2790-s001.doc]

**SCP19M24_aF**

**SCP19M24a1 1 ATGGCCTCATTGTCAGTAGAACTCTCACATTTTGTCCATTTTTTTTTGTTTCCTTTTACC 60**

**SCP19M24a2 1 ATGGCCTCATTGTCAGTAGAACTCTCACATTTTGTCCATTTTTTTT GTTTCCTTTTACC 59**

************************************************ ***************

**SCP19M24a1 61 AACTTGTTYTTTTTTGTATTTTTCAAAGAGCCAGKGYTACCCAACATTGGCAGAATCAGG 120**

**SCP19M24a2 60 GACTTCTTCTTTTTTGTACTTTTCAAAGAGCTAGTGCTACCCAACATTGGCAGAATCAGG 119**

****** **+********* ************ **+*+*************************

**SCP19M24a1 121 ATCCCAAAAGAGTGGACTAAACACGTCAAGATCATCMCTGATATCCCCCCCCCCCGAAGA 180**

**SCP19M24a2 120 ATCCCAAAAGAGTGGACTAAACACGTCAAGATCATCACTGATAT----CCCCCCCGAAGA 175**

**************************************+******* **************

***Pst*I**

**SCP19M24a1 181 ATGGGACGGTTTCCATGCATTTATCCTGCAGGATGAGTTGATGAGATGTGGCTCAGCTGG 240**

**SCP19M24a2 176 ATGGGGCGGTTTCCATGCATTTATCCTGCAGGATGAGTTGATGAGATGTGGCTCAGCTGG 235**

******* ********************************************************

**SCP19M24a1 241 GTGGATATTCATCTCACCTCTCTTCTTATCACCCCCCAAAAAAACTGATTGGGTTGATCT 300**

**SCP19M24a2 236 GTGGATATTCATCT---------------CACCCCCCAAAAAAACTGATCCGGCTGATCT 280**

**************** ******************** ** ********

**SCP19M24a1 301 CAAATTACAGTGCAGTGGGTGGCTTGTTCAGTGGGATGGCTTACGTGAGCGTTTGGGACC 360**

**SCP19M24a2 281 CAAATTACAGTGCGGTGGGTGGCTTGTTCAGTGGGATGGCTTACGTGAGCGTTTGGGACC 340**

*************** ************************************************

**SCP19M24_a1R**

***Mse*I**

**SCP19M24a1 361 AGTGACTTCCAATGAAATTACAATCAATATCGGAACTTGATTAATGAGTCTGAATTCTTY 420**

**SCP19M24a2 341 AGTGACTTCSAATGAAATTACAATCAATATTGGAACTTGACTAATGAGTGTGAATTCTTT 400**

***********+******************** ********* ******** *********+**

**SCP19M24_a2R**

**SCP19M24a1 421 ATTTGTCCCTTCAG 434**

**SCP19M24a2 401 ATTTGTCCCTTCA- 413**

*****************

**Fig. S1: Sequence alignment and primer binding sites for the polymorphic genome regions of SCAR marker SCP19M24.** The sequence of SCP19M24a2 is common in isolates of *Puccinia striiformis*. The SCP19M24a1 sequence in contrast is specific to aggressive strain 1 (PstS1) and 2 (PstS2) isolates. Sequences of AFLP restriction sites are shaded in grey. Binding sites of forward and reverse SCAR marker and sequencing primers for SCP19M24 alleles are indicated above and/or below the respective sequence with arrows in 5’ to 3’ direction. Numbers indicate sequence bp positions.

**SCP12M26_seqF**

**SCP12M26a1 1 ACAGATTCCGGTATAGWGTAGGCGACTCCTTTGAGATAAAATTTTTATGTATAGCCAGAG   60**

**SCP12M26a2 1 ACAGATTCCGGTATAGWGTAGGCGACTCCTTTGAGATAAAATTTTTATGTATAGCCAGAG   60**

****************************************************************

**SCP12M26a1 61 ACTGAGATGACTCATCATGAGCAGGAATGGCCACAATTTTGAGTGAGTCTAATCAACTCA  120**

**SCP12M26a2 61 ACTGAG----------ATGAGCATGAATGGCCGCAATTTTGAGTGAGTCTAATCAACTCA  110**

******** ******* ******** *****************************

***Mse*I**

**SCP12M26a1 121 CCATCACTCGGTTCACACCCAATTTAACCCAACTCGCTCAGGCATCAGGGGGAAGCTCAA  180**

**SCP12M26a2 111 ACACCACTCGGTTCACACCCAATTTAACCCAACTCACTCAGGTATCGAGGAGAAGCTCAA  170**

**** ******************************* ****** *** ** ***********

**SCP12M26_a1F**

**SCP12M26a1 181 TTACGCGTCAATCAGACCATCCTAAAACTAAGTTACGGATAGGAGCTTATCCTTCAAGAG  240**

**SCP12M26a2 171 TTGCGCGTCAATCAGACCA-----AAACTAAGTTACGCACC-GAGCTTATCCTTCAAGAT  224**

**** **************** ************* * *******************

**SCP12M26_a1F**

**SCP12M26a1 241 ATACTCTTTGATGTGGTGAGTGGTCAGAGCCGATCCATAGGTCTCTGCATGGAAAAGGGT  300**

**SCP12M26a2 225 ATACTCTTTGATGTGGTGGGTGGTCAGAGCCGATCCACAGGTCCCTGCATGGAAAAGGTC  284**

******************** ****************** ***** ****************

**SCP12M26a1 301 GCATGATGGCCAAACCTCTATTTGGGCCACTGGACTCA----------------------  338**

**SCP12M26a2 285 ACATGATGGCCAAACCTCTATCTGGGCCACTGGACTCAATATGCTCACTTGATGAAGTCC  344**

********************** ******************

**SCP12M26a1 339 ---TTTTTTGGGTCTGCGGGAAGGCGAAG-GGGTCGCTTTGCGACAGCCATTCCAACGGC  394**

**SCP12M26a2 345 CTATTCTTTGGGTCTGCGGGACGGCGTAGAGGGTCACTTTGCGAGAGCCATTTCAACAGC  404**

**** *************** **** ** ***** ******** ******* **** ****

***Pst*I**

**SCP12M26a1 395 TTCCCA------------------------------------------------------  400**

**SCP12M26a2 405 TTCCCACTGCAGACCAAACATAACTATGGCCCGCTTCGCTTCTTGGTGCGAGGTGTTGCA  464**

**********

***Mse*I**

**SCP12M26a1 ------------------------------------------------------------**

**SCP12M26a2 465 CCGGTCCTCGAGCGAAGCGAGAGGGACATATAAATTAACCTCATTGCTCTGCGTGACTTG  524**

**SCP12M26a1 ------------------------------------------------------------**

**SCP12M26a2 525 AGTCAATCTCACCCTCTTGGTGGAGAGGGTGTACATCCGCTCGTAATAAATACAAGCTCT  584**

**SCP12M26a1 ------------------------------------------------------------**

**SCP12M26a2 585 TGGAAAGTAGATTCCACGTTTTTATGCTGTAATCTTGATGATCTTCCCCTCCTTTCCATC  644**

**SCP12M26a1 ------------------------------------------------------------**

**SCP12M26a2 645 TACTCCTCTTATTTTTCCTGCCTTTAGTTCTCCTCCCATGTTCAAACCAAGTCATCAAAA  704**

***Mse*I *Mse*I**

**SCP12M26a1 ------------------------------------------------------------**

**SCP12M26a2 705 CCAGAATATTCCTTCAGCGCATTAACATTGTGACAAAATTAAGAAAGAAAACGAAGAGAG  764**

**SCP12M26a1 ------------------------------------------------------------**

**SCP12M26a2 765 AAAAAAGAACACAGAGAAAATAAATCTCCTCCAACTTCAAAAAACATTATAAAATCAAAA  824**

**SCP12M26a1 ------------------------------------------------------------**

**SCP12M26a2 825 CTAGGCGAGAGGAAAAATGGAGGAGACAGGAAGATGCKTTTGCATCTTGCGCTGATCTGG  884**

**SCP12M26a1 ------------------------------------------------------------**

**SCP12M26a2 885 GATACCATCACATGGAGAGAATGAGGACCAAAACAGAGGGAATCCTAAAAAACGTAAAAA  944**

**SCP12M26_a2F**

**SCP12M26a1 ------------------------------------------------------------**

**SCP12M26a2 945 AGCCCTCAAAATGGAGATTGAATCACGCGGGCTCAGATTCCTAATCTGTAAGCCTTGCTT 1004**

***Pst*I**

**SCP12M26a1 401 -----------------------------------------------CTGCAGACCCGGC  413**

**SCP12M26a2 1005 GCACCTACGCGCTCCTCGGCGGTGCCGCACGGCTTACCTAAGATTGACTGCAGACCCGGC 1064**

*****************

**SCP12M26a1 414 AGAAGGACTTGAGTTAGCCCAGCATAGGG-ACTGGGGG-AGCTTTTTTTTATGTATCTCT  471**

**SCP12M26a2 1065 AGAAGGACTTGAGTTAGCCCAGCACAGGGGACTGGGGGGAGCCTTTTTCTATGTATCTCG 1124**

************************** **** ******** *** ***** ************

***Mse*I**

**SCP12M26a1 472 CACAGCTCTGTTTTCATCATGGATCTAGGATCTTGATCAACCCGTGCTTTGAAATTAACC  531**

**SCP12M26a2 1125 CGCAGCTGTTTTTTCATCATGGATCTAGGCTCTCGATCAACCCGTGCTTTGAAATTAACC 1184**

*** ***** * ******************* *** ****************************

**SCP12M26a1 532 AATTGGACTTACGAGGCGTTTGGGTCAATAAAAAGTCCAGGACTCACGTCCCTTTCCACT  591**

**SCP12M26a2 1185 AATTGGACTTACGAGGTGTTTGGGTCAATATAGAGTCCGGGACTCACGTCTCTTTCCACT 1244**

****************** ************* * ***** *********** ***********

**SCP12M26_a2R**

**SCP12M26a1 592 GCCAGACTTTGCCCACTCACCTACAGCATTTGTCGAGCCATCATCCCGGTGCCAACCCCC  651**

**SCP12M26a2 1245 GCCAGAATTTGCCCACTCACCTACAGCATTTGTCGAGCCATCATCCCGGTGCMAMCCCCC 1304**

******** *********************************************+*+*******

**SCP12M26_a1R**

**SCP12M26a1 652 ACCCCTTGACACACTGTTACAACCCACTGTCTTCCATCCTACTGTTAGGCCTAGTCCACA  711**

**SCP12M26a2 1305 TCA----AGCCCCTTGACACATTCCACTGTCTTCCATCCTACTGTTAGGCCTATTCCACA 1360**

*** * * ** *** ****************************** ********

**SCP12M26_a1R**

**SCP12M26a1 712 CCATCACTACAAGAAAATTGGGAGCAACTGACCTCAGTTGACATGCAGCTCACTCTCATA  771**

**SCP12M26a2 1361 CCATC------------------------------------------------------- 1365**

*********

**SCP12M26a1 772 GAGCTCCAATTCAAGCTCAAGCCAACCTGTGTGTTTCAAGAGKGGTGCACCCTGGTGTGC  831**

**SCP12M26a2 ------------------------------------------------------------**

**SCP12M26a1 832 CTTTGTACATACAAGGGGATGAACAAGGTGTACCA-CACAGGAGGCTGGGAGCTTGGCTC  890**

**SCP12M26a2 ------------------------------------------------------------**

**SCP12M26a1 891  AAGCTCCAAGCATAGTGTGCTTTGAGCTCTAGCATGTCAACTGGGTTCAGTTGCACCCAA  950**

**SCP12M26a2 ------------------------------------------------------------**

**SCP12M26a1 951  TTTTCTTGCAGTGCATCTTGTGTTTTTGTGCACGCCACCCATTCTGTGTGATCTCTCCGT 1010**

**SCP12M26a2 1366  -----------------TTGTGTTTTTGCGTACGCCACCCATTCTGTGTGATCTCTCCGT 1408**

************* * *******************************

**SCP12M26a1 1011 TTTGGCCAAGGTAAGGATTGCAACAACCAGGTGATGGATGACTTGGAAAGAACCTCAAAT 1070**

**SCP12M26a2 1409 TTTGGTCAAGGTAAGGATTGCAACAGCCAGGTGATGGATGACTTGGAAAGAGCCTCCAAT 1468**

******* ******************* ************************* **** *****

**SCP12M26_seqR**

**SCP12M26a1 1071 CCGCTCGTYGGTTAGTTATCATCTCATGAAAACTATCTAAAAAAAA-CTGATGAATTGCC 1129**

**SCP12M26a2 1469 CCGCTCGTCGGTTAGTTATCATCTCATGGAAACTATCTAAAAAAAAACTGATGAATTGCC 1528**

**********+******************* ***************** ***************

**SCP12M26_seqR**

**SCP12M26a1 1130 CCTCAATAGCTTTGATCC 1147**

**SCP12M26a2 1529 CCTCAATAGCTTTGATCC 1546**

**********************

**Fig. S2: Sequence alignment and primer binding sites for the polymorphic genome regions of SCAR marker SCP12M26.** The sequence of SCP12M26a2 is common in isolates of *Puccinia striiformis*. The SCP12M26a1 sequence in contrast is lacking in aggressive strain 2 (PstS2) isolates but is present in both non-aggressive and aggressive PstS1 isolates. Sequences of AFLP restriction sites are shaded in grey. Binding sites of forward and reverse SCAR marker and sequencing primers for SCP12M26 alleles are indicated above and/or below the respective sequence with arrows in 5’ to 3’ direction. Numbers indicate sequence bp positions.

**Table S1: Comparison of the performance of SCAR markers in relation to that of AFLP markers.**

| **SCAR/AFLP marker** | **No. of isolates Compared** | **Agreement (in %)** | **Discrepancies (in%)** |
| --- | --- | --- | --- |
| P12M26 | 155 | 143 (92.3%) | 12 (77%) |
| P19M24 | 155 | 155 (100%) | 0 (0%) |
